# Analysis of the strategies used by iranian nurses for management of provided care for patients with COVID- 19: a qualitative study

**DOI:** 10.1186/s12912-023-01239-3

**Published:** 2023-03-31

**Authors:** Shokoh Varaei, Zhang Caihong, Zhang Siqi, Parvin Mahmoodi, Mehdi Rezaee, Ali Karimi Rezveh, Seydeh fatemeh mirbazegh

**Affiliations:** 1 Medical Surgical department, Nursing & midwifery school, Tehran University of medical sciences, Tehran, Iran; 2 International nursing school, Adult Nursing, Hainan Medical University, Hainan, China; 3 Hainan Medical University, Hainan, China; 4 Clinical Care Research Center, Nursing and Midwifery School, Kurdistan University of Medical Sciences, Sanandaj, Iran; 5 Tehran University of medical sciences, Tehran, Iran; 6 Seydeh fatemeh mirbazegh, Deputy of treatment, Tehran University of medical sciences, Tehran, Iran

**Keywords:** Nurses, Management strategies, COVID- 19, Iran

## Abstract

**Background:**

Nurses have been at the center of managing the COVID-19 outbreak through direct bedside care in respiratory, emergency and intensive care environments, managing hospital units, providing Covid-19 testing, vaccination and contact tracing. Thus, the present study aimed to analysis the strategies used by Iranian nurses for management of Provided Care for patients with COVID- 19.

**Methods:**

The present study was conducted based on the conventional content analysis method and Graneheim & Lundman approach. The participants included the nurses working in the COVID-19 wards and were recruited by purposeful sampling and based on inclusion criteria. The data were collected by conducting semi-structured, in-depth, one-to-one interviews until reaching data saturation.

**Results:**

In-depth interviews with 10 nurses represented four main categories and fifteen subcategories. Four main categories emerged in this study i.e. “justice in human resources management”, “The art and science of comprehensive nursing care”, “managers as agents of change in crisis” and “challenges and its management”.

**Conclusion:**

The nurses’ experiences of management strategies showed that paying attention to the financial, psychological, educational, equipment needs of nurses and maintaining their safety make the suitable environment for providing high quality care for patients with covid-19.

## Introduction

A pandemic is the simultaneous global transmission of an emerging and re-emerging infectious disease epidemic, which affects large numbers of people worldwide and often results in significant mortality, social and economic disruptions [1].

The World Health Organization used the word pandemic for the Covid-19 disease due to its spread on March 11th 2020 [2]. COVID-19 was first detected in two people residing in Qom, a metropolis in Iran on February 19, 2020 according to the Iran’s Ministry of Health [3]. More cases were reported in other cities of Iran including Tehran which is the capital of Iran, Rasht a city in northern Iran, and also in the city of Shiraz, Fars province [4].

Patients with symptoms such as fever, dry coughs and respiratory distress and those with more severe forms of the illness are usually hospitalized for further treatment. The World Health Organization has released guidelines on how countries should prepare to deal with the COVID-19 [5, 6]. A state of emergency was declared, large gatherings were banned, fitness centers and restaurants were closed, and all elective surgeries were cancelled [7]. Nurses are considered as one of the key components in managing hospitals and the health care system and responsible for implementation of these measures. However, health care personnel, especially nurses who care for suspected or patients with confirmed corona virus at the hospitals are at risk of infection and the related challenges and consequences [5, 6]. the healthcare system and the role of healthcare workers as a vital elements of the healthcare system are responsible regarding the life of people in a pandemic. They may experience fell of being torn between professional duty and fear of being infected, or infecting others [8].

Nurses ought to look after people experiencing severe forms of the disease and should do so for long hours using protective equipment. But these individuals may have the fear of transmitting the virus to their family and friends [4, 6]. Nurses who cared for MERS-CoV^1^ patients in Saudi Arabia also experienced psychological trauma owing to the infection of their colleagues and deaths of their patients [9]. In addition, they felt ethical pressure because they were obligated to provide care despite the threat to their safety [10]. A study of the overall, holistic experience including not only the negative but also the positive experiences and support factors is needed.

In such cases, when a deep understanding of a specific phenomenon is required, qualitative research is recommended [11]. Indeed, there is a growing recognition for the important role played by qualitative research and its usefulness in many fields, including an outbreak of a contagious disease or emergency situations [12]. In order to achieve the management strategies used by Iranian nurses caring of patient with Covid-19, qualitative approach is the most suitable methodology.

Considering that, limited studies regarding the experiences of nurses in the covid-19 epidemic has been done separately from other health Care workers^2^ until now, and qualitative studies have been conducted on the experience of nurses in caring for patients with covid-19 in countries which are different in terms of social, economic, cultural status, care system, diagnostic and treatment facilities.

Content analysis approach was used in order to understand the strategies for management of provided care for patients with COVID- 19. This pandemic has brought significant pressure on nurses globally but especially Iranian nurses. How Iranian nurses could manage the provided care for patients with covid-19 in the situations of shortage of facilities and nurses; fear of infecting themselves and their families; a newborn disease with little knowledge about it and dealing with specific challenges of patients with covid-19 and crowded hospitals. This study will be a basic resource to establish a safer healthcare system that can protect both patients and healthcare providers and respond quickly and systematically to similar situations in the future.

## Methods

### Study design and participants

Conventional content analysis approach of the Graneheim & Lundman method was used to explore what kind of strategies used for management of cares provided for patients with COVID- 19 by Iranian nurses [13]. Iranian nurses working in the COVID − 19 departments who would like to participate in the study and share their experiences were selected through purposeful sampling method as participants. Inclusion criteria included working in the covid-19 department for at least 1 month as a nurse and willing to participate in the study. Exclusion criteria included willing to leave the study at any stage of the study. The first participant was a key informant who had rich experiences of caring of patients with covid-19. She was a 32 years old clinical nurse with 11 months’ experience of working in covid-19 wards. Sampling continued until data saturation occurred. Ten nurses participated in the study. Demographic information of the participants is presented in Table 1. The guideline of consolidated criteria for reporting qualitative research (COREQ) was used for providing this manuscript.

**Table 1:** Demographic information of Iranian participants

Variables	Subcategories	NO of participants
Age	20–30	1
	31–40	4
	41–50	4
	More than 50	1
Gender	Female	10
	Male	0
Marriage status	Married	7
	Single	3
	Separated or deceased	0
work experience (Years)	1–10	3
	11–20	5
	21–30	2
Time to Attend COVID-19	1–6	6
	7–11	4
Position	Nurse	5
	Head nurse	3
	Supervisor Educational	0
	Clinical Supervisor	1
	Matron	1

### Data collection instruments

Data were collected using semi-structured interviews. The interviews usually began with a general question: “what is the strategies for management of provided care for patients with COVID- 19 by Iranian Nurses?” Or “tell me the story of one day of your experience in COVID- 19 department. Additionally, further explanations were also obtained based on responses of the participants and by asking complementary probing questions such as: “please explain more…. Or what do you mean about…?” (Table 2).

**Table 2:** General questions which were asked of nurses during interviews

1	what is the strategies for management of provided care for patients with COVID- 19 by Iranian Nurses?
2	tell me the story of one day of your experience in COVID- 19 department.
3	What was the specific work that the managers (nurses) of hospital undertook in response to COVID-19
4	Was there any specific equipment needed for this negative pressure ward?
5	did you have a training program for nursing staff and how was it undertaken?
6	What evidence did you refer to make the emergency plans?
7	have you encountered any difficulties or challenges in managing human resources? How did you manage them?
8	At the time of the outbreak, most of literature was actually not up to dated. So what are the main criteria to refer the literatures?
9	what measures was taken for nurses and patients who might have psychological problems?
10	was the human resource and equipment’s sufficient? Was there a shortage of human resource?

Mutual agreement of interviewees and researchers determined the time and place of the interview sessions. All of interviews were done in the hospital. The interview time was 30 to 85 min and on average 38 min for each interview. A digital device was used for recording the interviews. The interviews were continued until data saturation, where categories and subcategories were completed and no new category was obtained [14].

### Ethical considerations

The Ethics Committee of Tehran University of Medical Sciences (TUMS) approved the study protocol (Ethical code = IR.TUMS.FNM.REC.1399.132). Ethical considerations in this study included explaining the importance, objectives and methods, optional participation in the study, recording the interviews, maintaining data confidentiality at all stages, getting written consent, and mutual decision about the time and place of the interview.

### Data analysis

Content analysis approach and constant comparative analysis was used for data analysis. Each interview was considered as a unit of analysis. The interviews were recorded and transcribed verbatim and read repeatedly. Then, the data were broken down into units of meaning that were extracted from the statements and labeled with conceptual names. Then codes were compared based on similarities and differences and grouped into categories. Each subcategory with similar mean was grouped as categories and categories were grouped as main categories [14]. MAXQDA 10 was used to manage the textual data.

### Trustworthiness

This study applied the criteria suggested by Guba and Lincoln to evaluate the credibility of the.

data [15]. They rely on four general criteria in their approach to trustworthiness. These are credibility, transferability, dependability, and confirmability. The prolonged engagement with the participants during the interview period helped to establish trust, better understanding and a close relationship with the participants. Moreover, analytic categories, interpretations, and conclusions were tested using member checks. The prolonged engagement with the participants during the interview period and member-checking were used for credibility. Detailed descriptions of contexts and participants were used for transferability. Inquiry audit was used for dependability. Triangulation was used for confirmability.

## Results

The analysis of the data obtained from the interviews led to the extraction of the main category “managers as key element to overcoming the crisis”. In total, 4 categories and 15 main subcategories were extracted from the interviews which are as follows (Table 3).

**Table 3:** Main Category, Category and subcategories extracted from the interviews of Iranian nurses

Main Category	Category	Main Subcategory
Managers as key element to overcoming the crisis	1.Justice in human resources management	1.1 Motivating employees
		1.2 Providing protection and welfare facilities to employees
		1.3 Human resource management mechanisms
	2. The art and science of comprehensive nursing care	2.1 Patient education
		2.2 Nursing precautions in oxygen therapy of COVID-19 patients
		2.3 Team work
		2.4 Sacrifice of nurses in the fight against COVID-19
	3. Managers as agents of change in crisis	3.1 Changes in the management of hospitalized COVID-19 patients’
		3.2 Strengthening physical infrastructure of hospital
		3.3 Management measures to provide high quality care
	4. Challenges and its management	4.1 Psychological consequences of Covid-19 on nurses
		4.2 Managers’ concern regarding nurses’ intention to leave profession
		4.3 Challenges related to PPE
		4.4 Challenges caused by the emergence of Covid-19
		4.5 The challenge related to the rejecting of nurses to being appreciated

### Justice in human resources management

This category includes 4 main subcategories include of “Motivating employees”, “Providing protection and welfare facilities to employees ”, “Justice-Based leadership” and “Human resources management mechanisms”.

#### Motivating employees

Managers thought of measures as follow to motivate their employees.

##### Holding motivational webinars and resident psychiatrist for visits

At the beginning of the pandemic, nursing managers requested to hold motivational seminars for nurses. During these seminars, psychiatrist emphasized on job duties, receiving divine rewards and the vital role of nurses to save the society. A psychiatrist was at the hospital so that those nurses who felt depressed or frustrated could refer to him. A nurse said in this regard:

“The psychiatrist held a motivational seminar for colleagues. It was really effective in reducing the stress of our staff.”

##### Increasing nurses’ salary

Another support measure of the mangers was financial encouragement of nurses in front of difficult work and overtime. It was one of the factors of not leaving covid-19 wards. A nurse said:

“In a situation where everyone was working away, our work doubled. The increase in the payment of the nurses working in the quarantine wards increased their motivation.”

##### Mandatory leave for nurses

In order to calm the panicked staff, the head nurses gave them permission to spend a few days with their families. Sometimes, few nurses were malingered to leave quarantine ward for few days. Head nurses did not resist these nurses, but took PCR for COVID-19 from them and they were given 3 days of rest at home until the results were ready. A head nurse said regarding this:

“We were trying to give a few offs and to 7 days of leave during the month so that they can visit their families and relax to rejuvenate themselves mentally.”

##### Volunteer managers, nurses and physicians for working in quarantine wards as role models

Matron, a lung specialist, a nurse who volunteered to work in the quarantine wards were all a role model for other nurses. A nurse said in this regard:

“When we were told to work in the ward of Covid-19 patients for the first time, we saw the matron who came and got dressed; she took care of patients with COVID-19. We saw lung specialists working voluntarily. We would follow them as a role model.”

##### Training and prompting the awareness of nurses with the latest findings on the COVID-19 pandemic

Emerging nature of Covid-19 highlighted the necessity of training in physicians and nurses. The main source of the initial fear of the HCWs was ignorance. Therefore, nursing managers held training courses to create a sense of empowerment in employees. WhatsApp virtual and face-to-face communication channels were created between managers and supervisors to exchange protocols and notifications. A nurse said the following about the method and content of training:

“Acute respiratory care and all notifications and protocols were shared with nurses through WhatsApp channels.”

##### Sick leave and free medicine for infected HCWs

As soon as each nurse was confirmed to be infected, she was given sick leave and free medicine. During the course of his illness, his/her recovery was constantly monitored. In this way, the hospital instilled in its personnel the feeling of being part of a family.

“In order to feel that they are being treated like family here, a common approach was taken with the staff. That is, I will get PCR-COVID 19. I will go home for three days. If I am infected, they will give me medicine for free.”

##### Daily leadership rounding of the COVID-19 wards and listening to the HCWs’ concerns

The daily leadership rounding created a close relationship between the nurses and managers so that they are aware of the deficiencies and problems and take necessary measures to solve them as quickly as possible. A nurse said in this regard:

“Management team come for leadership round every day, ask about the deficiencies, problems and try to solve the problems on time and as soon as possible.”

#### Providing protection and welfare facilities to employees

Another thing that aroused the feeling of trust in the organization in the nurses was providing protection and welfare facilities to employees. Three of them are mentioned below.

##### Providing high-quality and sufficient medical equipments and PPE^3^

Part of the nurses’ expectations was met through the provision of the most expensive equipment, including PPE, ventilators, and NIV despite the sanctions. Equipment shortage was managed by controlling and counting the number of equipment in each shift. All these measures ensured that HCWs did not experience a shortage of equipment during the pandemic. A nurse said:

“The Ministry of Health did the best regarding providing PPE, ventilators and NIV masks, which are very expensive and under sanction.”

##### Hotel accommodations for HCWs during COVID-19 pandemic

One of the main and most common concerns of nurses during the pandemic was the fear of transmitting the virus to their families. Managers booked a hotel close to the hospital so that if the nurses wanted, they could rest in the hotel instead of going home. A nurse said in this regard:

“A hotel was booked by hospitals’ managers for us so that we could stay in quarantine and did not come back home at all.”

##### Providing facilities for taking shower and disinfection of HCWs before leaving the hospital

The hospital managers took measures that HCWs could take shower and disinfected at the end of each shift and before entering their house. A nurse said:

“We were told that you must take a shower at hospital and then go home.”

#### Human resource management mechanisms

At the beginning of the pandemic, shortage of HCWs were due to the high rate of morbidity and hospitalization of patients. Therefore, managers thought of measures to compensate for this deficiency.

##### Insecure employment contracts for overcoming nurses shortages

In order to compensate the shortage of nurses and their long working hours of the nurses, the managers signed contracts with the nurses characterized by reduction in the contract period to 89 days with the possibility of extension if necessary. This action led to the entry of a large number of unemployed nurses into the field of work.

“To compensate the shortage of nurses, sign an 89-day contract with nurses”.

##### Employing operating room and anesthesia nurses to work in the quarantine wards

Since elective surgeries were not performed for a long time during the pandemic, the nurses of the operating room and anesthesiologists were off work. For this reason, they were used in the quarantine wards to compensate the shortage of nurses and to reduce the work pressure of the nurses working in quarantine wards. A supervisors said:

“If we allocated 10 to 12 patients with COVID-19 patients to a nurse, the nurse will not be able to take care of them. Employing anesthesia and operating room nurses made each nurse have 5 to 6 patients.”

##### Calling for non-medical volunteer people to perform the basic needs of COVID-19 patients

A number of hospitals also announced a public call to accept non-medical volunteers. These forces were trained to do oxygen therapy, feeding and bedside elevation. A nurse said about this:

“We tried to teach volunteers oxygen therapy, feeding, and elevating bed side and the head of the beds.”

### The art and science of comprehensive nursing care

Treating the COVID-19 of patients was not the only goal of nurses. Nurses are creative in taking care of COVID-19 patients.

#### Patient education

Nurses entrust part of the care to the patient by educating them. They stimulate patients’ cooperation in care through improving their awareness.

##### Educating regarding medication regimen and their complications to patients

During hospitalization, the patient was given the latest information about the way to take the medicine in order to prevent complications. A nurse said in this regard:

“For example, it was very difficult for patients to take hydroxychlorine tablets because it had very severe gastrointestinal complications. We taught them what to do so that these complications reduced.”

##### Training patients to prevent others from getting infected during hospitalization and discharge

In order to prevent the patient from re-infecting himself and infecting others, training was given to patients both during hospitalization and before discharge regarding quarantine, social distancing, disinfection, etc. A nurse said about such trainings:

“We focused a lot on prevention so that they prevent from getting COVID-19 again. For example, we taught them to keep a distance of 2 meters, we taught the patient’s family and the patient himself to keep the distance, use a mask and wash their hands frequently.”

##### Educating incentive spirometry to rehabilitate the patients’ lungs

In order to rehabilitate the lungs, patients were given incentive spirometry. During hospitalization, he was taught how to use incentive spirometry and its favorable consequences. A nurse said in this regard:

“We used to teach patients how to use incentive spirometry at home for lung rehabilitation.”

##### Educating the patient in terms of diet (abundant liquids and high protein)

Considering the weakness and dehydration of the Covid-19 patients and the importance of nutrition in their recovery process, all educational tips related to nutrition were given to the patients. A Nurse said:

“We taught the patients to drink more water and fluids, eat high-protein, carbohydrate and vitamin C foods.”

##### Educating oxygen therapy at home

A number of patients had to use oxygen at home after discharge from the hospital. Therefore, all the precautions related to the use of oxygen were taught to the patients. A nurse said in this regard:

“I was explaining to him that the normal range of blood oxygen is, for example, 92 to 97%. For example, your saturation is 96, so you do not need to receive oxygen.”

##### Educating respiratory warning signs and the necessity to refer to the hospital

When the patients were discharged, since there was a possibility of their condition getting worse, they were taught the symptoms that indicated the need to receive hospital services. A nurse said about this:

“If they feel short of breath and cannot control themselves in terms of breathing, they should go to the hospital.”

#### Nursing precautions in oxygen therapy of COVID-19 patients

After a while, nurses gained a lot of experience about the tips of oxygen therapy for COVID-19 patients.

##### Step-by-step application of oxygen therapy equipment based on the patient’s condition and response

Nurses tried to raise blood oxygen saturation with the lowest amount of oxygen support, so they used nasal cannula, simple mask, mask with reserve bag and NIV mask respectively. A nurse said in this regard:

“We were trying to use, nasal, then mask, reserve bag mask and NIV masks.”

##### Nurses’ efforts to tolerate NIV mask by the patient despite their resistance

The nature of the NIV mask, the high pressure of the oxygen coming out of it, and the need to coordinate the patient’s breathing with the mask, etc., often made patients to refrain from using it. To create a sense of trust and cooperation of the patient, they stayed with the patient for a long time to convey the feeling of the nurse’s availability. The patient was NPO 1 h before and after the NIV mask. They taught the patient how to coordinate their breathing with the mask. They advised not to talk while using the mask and to call the nurse by hitting the bad side. They protected pressure points on the nose and face of the patients with cotton. A nurse said in this regard:

“It was very difficult for the patient to tolerate the NIV mask. We used to calm the patient down, nurses were standing over the patient’s head for more than half an hour and 40 minutes, in order to calm the patient down and regulate their breathing.”

##### Emphasis on to not incubating patients with COVID-19

Nurses are used to prevent patients from intubation because experience shows that it is hard to wean patients off a ventilator. A nurse said about this:

“Usually, we emphasis on not incubate the patient, because if patient was incubated, then weaning off a ventilator becomes difficult.”

##### Confronting with patients’ psychological dependence on receiving oxygen

Hospitalized patients were psychologically dependent on receiving oxygen. To deal with this dependence, they gradually reduced the amount of oxygen received by the patients and by educating the normal range of saturation; they convinced the patient that he/she does not need to receive oxygen. A nurse said in this regard:

“Giving oxygen to the patient has made them very dependent on oxygen and fear that if they don’t get oxygen they might asphyxia.”

#### Team work

One of the positive aspects of the COVID-19 pandemic was the strengthening of teamwork among HCWs.

##### Exchange of information, empathy and more cooperation between HCWs in the pandemic era

Due to the daily training of the nurses by the expert physicians, the relationship between them improved. On the other hand, it increased the correlation between all the HCWs for the purpose of better treatment of the patient and not infecting HCWs. A nurse said in this regard:

“They cooperate at all management levels of the hospital. Everyone is trying to take a burden off everyone else’s shoulders.”

##### Cooperation of nurses and physicians with pharmaceutical companies in carrying out research projects regarding Covid-19

Covid-19 as emerging diseases made it necessary to carry out research projects to find out more about COVID-19. For this reason, nurses cooperated with doctors and pharmaceutical companies. A nurse said in this regard:

“Many research projects were conducted regarding CovID-19. Each Physician cooperate with a nurse and pharmaceutical company to conduct the research.”

#### Sacrifice of nurses in the fight against COVID-19

COVID-19 pandemic doubled the difficulty of nursing.

##### Telecommuting of other jobs versus the harder work of nurses in the pandemic

Importance of nurses in crisis management was more than other jobs, because nurses were in contact with patients with Covid-19 during long 24-hour shifts.

During this time, other businesses took rest and Telecommuting, while the vacations of the HCWs were canceled and their work time were increased. A Nurse said in this regard:

“The working hours of other jobs were reduced during pandemic, while nurses didn’t have holidays and still working hard.”

##### Caring with love of COVID-19 patients

Nurses reported that patients’ hemodynamic status became more stable after being calmed down by them. Resuscitation was carried out for a long time for these patients in the hope of saving not only the life of a patient but also of a family. A Nurse said in this regard:

“The more we explained about the patient’s illness and condition, the less stressed he/she was. The condition of the patient was getting better in terms of general condition.”

### Managers as agents of change in crisis

Managers played a prominent role in the successful control of the Covid-19 crisis.

#### Changes in the management of hospitalized COVID-19 patients’

Due to the lack of previous experience in dealing with such a pandemic, the passage of time was necessary to gain experience on how to manage these patients.

##### Limiting the COVID-19 length of hospital stay

At the beginning of the pandemic, every symptomatic client was hospitalized for 14 days which increases the risk of hospital infections. Later, patients were discharged if fever and respiratory distress were controlled. A nurse said in this regard:

“We taught the patients to go home and continue the treatment at home. They become more depressed in the hospital.”

##### Changing the admission procedure

At the beginning of the pandemic, all infected people were admitted to the hospital, even with the least symptoms. After a while, only patients with extensive lung involvement and needing hospital services were admitted. A nurse said in this regard:

“Patient with the smallest symptoms of COVID-19 was hospitalized, in order to identify the symptoms. After a while, just patients who were more involved and needed hospital care, were hospitalized.”

#### Strengthening physical infrastructure of hospital

Due to the contagious nature and involvement of the respiratory system in COVID-19patients’, required the provision of equipment and changes in the physical space of the COVID-19 centers.

##### Providing the necessary equipment for the COVID-19 centers

Due to the spread of this virus through surfaces, most hospitals bought a separate CT scanner to be used only for patients with COVID-19. Other equipment provided for the care of these patients included ventilators, serum pumps, and NIV masks. A Nurse said in this regard:

“Ventilators, serum pumps, NIV masks, oxygen booster tanks and support equipment for employees such as masks, gown, and shields were provided despite the crisis and shortage”.

##### Evacuation of wards and their physical separation by a wall from other non-COVID-19 departments

A number of wards were evacuated to provide physical infrastructure and human resources. A Nurse said in this regard:

“COVID-19 wards were separated from vulnerable departments such as blood and oncology, dialysis, etc. by a wall in terms of physical distance.”

#### Management measures to provide high quality care

Providing high-quality care is the mission of all centers for hospitalized COVID-19 patients.

To achieve this goal, managers took some measures.

##### Forming a respiratory team to daily control the settings of the ventilators

In order to increase the accuracy of ventilator settings, a team called the respiratory team was formed in the hospital, which consisted of experienced nurses in the field of working with ventilators. A Nurse said in this regard:

“The breathing team was formed in the hospital, which checked the settings of all the ventilators of patients with Covid-19 daily and made the necessary changes if necessary.”

##### Frequent control of the quality and accuracy of nursing care by nursing managers during rounds

Long-term use of masks by nurses led to their hypoxia and increased possible error rates. Therefore, the nurses’ work was controlled several times by the supervisor, nurse, and head nurse in order to prevent errors. A Nurse said in this regard:

“Frequent presence of the nursing manager and supervisors in the wards to control the protocols would increase the quality of care.”

##### Employing skilled nurses and physicians in the COVID-19 wards

Quarantine wards were not a place for new recruits or medical and nursing students to train. Because the unknown nature of this disease required elite physicians and nurses to recognize this disease in a short time and provide high quality care. A nurse said in this regard:

“Only expert nurses, physicians with high experience and senior year residents worked in the quarantine wards. If there were no elite people, we would not have been able to control this pandemic.”

##### Creating common protocols for the treatment and care of COVID-19 patients

Based on the results of the latest studies of the Ministry of Health, in order to integrate the treatment and care of patients with Covid-19, they were designing protocols. These protocols were prepared and installed in the quarantine wards regarding oxygen therapy, fluid therapy, corticosteroid therapy and the type of antibiotics. A nurse said in this regard:

“Different professors would design and review the protocols together in the meetings and we would implement them in the wards.”

##### Collecting information on how other countries are reacting to COVID-19

It was very helpful to use information and how countries that have been involved in this disease for a longer time reacting to COVID-19. A nurse said in this regard:

“The first task in facing the Covid-19 crisis was to collect information from all over the world on how to manage it.”

##### Allocation of a team in the hospital to answer the concerns of patients’ families in person and by phone call

Since hospitalized patients with COVID-19 were prohibited from visiting, therefore, in order to prevent many families from visiting the hospital, a room in the hospital where several nurses were working was established under the title of public relations. These nurses used to receive the history of the patients by phone from the wards on a daily basis, and if the families called, the needs of the patients and the general condition were announced. A nurse said in this regard:

“The public relations of the hospital appointed two nurses who would take the history of the patient from us, how the patient is now and what he needs, then he would call and tell the family.”

### Challenges and its management

Nurses experienced many challenges while caring for patients with Covid-19.

#### Psychological consequences of Covid-19 on nurses

Nurses were under pressure from their families, the work environment and observing the high mortality rate of patients and colleagues.

##### Feeling of powerless in patients treatment

The unpredictable process of the COVID-19 disease caused frustration and powerlessness in the nurses. For example, Patients who were in the process of recovery, suddenly their saturation were decreased and finally they were intubated.

“What I remember about my mental state at that time was that I really didn’t want to see that i can’t do anything for patients.”

##### High daily mortality rate

At the peak of Covid-19, the high mortality rate of patients caused a lot of panic among nurses. A nurse said in this regard:

“I wish I didn’t wake up in the morning. I was so emotionally stressed, as every day you come; you see a series of families of patients are crying.”

##### Psychological tensions of nurses’ families

The nurses’ families expressing concern and crying when the nurse is on shift at the bedside of the COVID-19 patients doubled the nurses’ tension. A nurse said in this regard:

“My family told me that you should go write your resignation and come back home. When I came home from my shift, I saw that my mother had cried so much the night before when I was there.”

##### Morbidity and mortality of nurses

Nurses were also infected with covid-19 at a high rate. However, a small number of nurses died due to COVID-19. Nurse said in this regard:

“It was very upsetting that two of our colleagues died.”

##### Feeling frustrated

The nurses participating in this study likened working in the covid-19 wards to fighting on a war front because they did not know what might happen. I can clearly feel the decrease in mood compared to before the pandemic. Nurse said in this regard:

“Before of the pandemic, I used to go to the hospital every morning when I got up, so I was happy, but in this pandemic, I woke up many days and said to myself, I wish I couldn’t go to work.”

##### Afraid of nurses

Panic and fear were seen in all nursing groups at the beginning of the pandemic. Ignorance, high rate of transmission, lethality, lack of specific treatment and most importantly the possibility of transmission to family members were the reasons for fear. A matron said:

“The day I told my colleagues that they should work in the quarantine wards, I faced terrible resistance. All of them were upset and crying.”

#### Managers’ concern regarding nurses’ intention to leave profession

The nurses’ concern about the possibility of the transmission the virus to their families, not visiting their families for a long time by nurses, and the resignation of a number of nurses worried the managers. Administrators were worried that nurses would leave hospitals.

##### Resignation of a number of nurses

At the beginning of the pandemic, the first reaction of many nurses was to resign under the pressure of their families. The main reason for nurses to resign was the fear of death for themselves and their families. A nurse said in this regard:

“At the beginning, half of the staff announced that we are going to resign. We don’t want to put ourselves and our families at risk.”

##### Nurses’ concern regarding the possibility of transmission the virus to their families

One of the main concerns of the nurses was the possibility of being a carrier and transferring it to their family members. A nurse said in this regard:

“At the beginning of COVID-19, we were all stressed, not to be carriers ourselves, but to pass it on to our families.”

##### Not visiting the family by the nurse for a long time

The nurses had not seen their families for a long time. After making sure that the PCR test for COVID-19 was negative and consulting with an infectiologist, they visited their family at a long distance. A nurse said in this regard:

“It was almost 4 months that I didn’t see my family because I was worried that I would transmit to them.”

#### Challenges related to PPE

Despite the protection made by PPE for nurses against COVID-19, it was associated with several challenges.

##### Difficulty of tolerating PPE equipment for a long time

Around the lips, on the nose and face of the nurses and their body would suffer from eczema. It was due to long-term use of the mask, sensitivity and sweating. A nurse said in this regard:

“Isolation clothes are made of plastic, so nurses had eczema on their bodies and hands in the hot weather. We used tropical ointment for relieve.”

##### PPE as barrier communication between the patient and the nurse

Another problems created by PPE were the non-identification of nurses by patients, the impossibility of lip-reading for patients with hearing loss, and increasing the stress of patients. A nurse said in this regard:

“The big problem we had was our clothes and masks. Patients couldn’t see our faces, so they couldn’t recognize us. the patients who were hearing loss, they couldn’t lip-reading.”

##### Patients become anxious when seeing PPE

Seeing the PPE increased the patients’ anxiety and gave them the feeling of having a dangerous disease. A nurse said in this regard:

“The patients were very stressed when they saw us in these clothes.”

##### Nurses’ unscientific information about the quantity and quality of PPEs

At the beginning of the pandemic, nurses were not sure about the quantity and quality of PPE due to their lack of knowledge. Nurse said in this regard:

“We didn’t know at all that does these PPE have enough efficacies?”

#### Challenges caused by the emergence of Covid-19

The emergence of covid-19 caused frequent changes in data about the transmission method, treatment protocols, and disagreements among physicians about the treatment method.

##### Frequent changes in data regarding methods of transmission of Covid-19

HCWs’ precautions to prevent infection were based on the latest data on the ways of transmission of the COVID-19, which were changing rapidly. A nurse said in this regard:

“The information changed day by day.”

##### Continuous changes in treatment protocols for COVID-19

The continuous change of the lines of treatment of COVID-19 challenged the nurses. For example, the new medicine was prepared with a long time delay and the complications of the new medicine were unknown. A nurse said in this regard:

“Protocols were changing every day, it was very problematic for us.”

##### Physicians disagreement on how to treat COVID-19 in the early stages of the pandemic

At the beginning of the pandemic, there was no consensus among physicians for the medical treatment of COVID-19, and sometimes physicians acted arbitrarily. A nurse said in this regard:

“All these differences in the opinions of the physicians caused disunity.”

##### Vary responsible physicians for visiting patients in the COVID-19 ward

Changing the physicians responsible for the visits of Covid-19 patients created challenges for the nurses. These challenges include of unfamiliarity with routine treatment, anxiety about exposure and infection, and complaints about the lack of PPE. A nurse said in this regard:

“The nursing team were fix in each shift, but the physicians who came for visit changed every day, so they were not aware of the routines.”

#### The challenge related to the rejecting of nurses to being appreciated

Nurses experienced a range of interactions with others during pandemic.

##### Pressuring nurses by their families to resign

Some nurses received a lot of pressure from their families to resign. A nurse said in this regard:

“When I told my parent that I am going to work in COVID-19 Center, they asked me to go hospital and write your resignation.”

##### Motivating the nurse by her family to work in the COVID-19 ward

A group of nurses was not pressured by the family, but the family became a motivating factor to work in the COVID-19 ward. A Nurse said in this regard:

“The biggest favor my family has done for me is being very supportive.”

##### People and media appreciate the nurses after the pandemic

The society’s view of the HCWs changed after the covid-19 pandemic. A nurse said in this regard:

“Receiving a message of acknowledgment by nurses from friends and patients is a pleasant feeling.”

##### Rejection of nurses engaged in COVID-19 by society and non-COVID HCWS

Family members, relatives, neighbors, supervisors and hospital personnel who worked in non-Covid-19 wards avoided the nurse in the Covid-19 ward in order not to get infected. A nurse said in this regard:

“I could really see that we were rejected and a wall was drawn in front of our department. The colleagues from other departments were distancing themselves from us and we were very upset.”

## Discussion

This study aimed to investigate the strategies used by Iranian nurses for management of patients with COVID- 19. According the results of this study managers as key element tried to overcoming the crisis through applying justice in human resources, providing comprehensive nursing care, making change and managing the challenges. Our results were categorized into four main categories, which will be discussed in the following sections.

Human resources are the most important part of an institution, especially in a crisis. The results of this study showed that taking measures by managers to create justice in human resource management, especially in the shock phase of facing the Covid-19 crisis, was able to keep nurses in hospitals. In the first days of the beginning of the pandemic, most of the nurses were shocked, crying and worried about their infection and transmission to their families. Many participants in this study reported that the thought of leaving the service had crossed their minds.

In fact, epidemic diseases can have a significant impact on nurses whose presence is necessary for providing health care services [16]. Pandemic diseases exacerbate nurses’ stress as they are faced with severe emotional, physical, and cognitive demands and must adapt to them [17, 18]. The result of this study showed that COVID-19 pandemic has the same effects on nurses. In the frontline of care provision they face pain, death, and moral dilemmas.

Therefore, nursing managers participating in this study by holding motivational webinars, increasing nurses salary, daily round of quarantine departments, working alongside other nurses in the quarantine wards as a role model and following up on symptomatic personnel resting at home made nurses motivated.

In addition, the shortage of human resources and lack of equipment make their work even more exhausting due to imposing a high workload and exposing them to potentially health threatening conditions [19].

The nursing managers proved the importance of the health of the nurses and their families by providing high quality and sufficient PPE and providing a hotel for the nurses to stay.

Also, Perron and Gagnon [20] described nurses as “foot soldiers” who are sent to a war without proper equipment (or even with no equipment), sufficient information, and adequate human forces and physical resources, and even without adequate support and compensation. Other studies have also referred to nurses as “war heroes” [21, 22].

A justice-based approach with all personnel, from the dean of the hospital to the nurse, regarding the quality of the protective equipment used, how to grant leave work when symptoms occur, dividing patients according to their difficulty and care needs among the nurses, and immediate solving the problems of the nurses in the quarantine wards, is reminding the equality of personnel from the organization’s view.

One of the measures taken to compensate for the shortage of nurses in Iran was signing 89-day contracts with unemployed nurses. This measure partially solved the need of the organization, but unfortunately, after the end of the covid-19 couriers, they did not renew the contract with the nurses.

Regarding cultural perceptions Foster [23] stated that “all of the efforts to maintain a culture of safety and prevent harm have a common denominator: They’re dependent on the hands, hearts, and minds of the staff”. So, during the COVID-19 life-threatening conditions, nurses feeled more responsible to provide the suitable care, but it can vary based on cultural outlooks of nations.

Iran is an Islamic country where nurses take care of patients with Islamic culture and religion. In addition to the physical needs of the patients, with the importance of the emotional and religious needs of the quarantined patients, the hemodynamic and breathing conditions of the patients became more stable. Considering the adverse impact of observing sick patients by patients with a more favorable general condition, Iranian nurses determined the patient’s room based on their general condition. Another Iranian nurses’ creative were placing intubated patients close to the nursing station.

Therefore, nurses from different social and cultural bases have diverse ethical and religious knowledge which may impact their care that they provide to the patients [24, 25].

Despite all the stressful and life-threatening conditions of the pandemic for nurses, telecommuting other jobs, nurses sacrificed themselves to take care of Covid-19 patients and took their lives in their hands.

Being in the frontline position deeply undermined nurses’ professional self, which led most of them to be more likely to demonstrate self-sacrifice (i.e., when they were asked to work regardless of their health and exhaustion) [26]. A previous research has shown that self-sacrifice is an intrinsic coping strategy for nurses to overcome tough situations, when they do not feel sufficiently supported [27].

One of the interesting experiences of the participants of this study was the transformation of threats into opportunities. They reported a significant improvement in teamwork between HCWs and crisis management during the pandemic.

This result was not in line with a study which reported challenges between various healthcare specialties in team working in different provinces of China [8]. This difference could be due to the difference in the context of the studies.

Nursing managers controlled the quality of nursing services and reduced the burden on hospitals with measures such as formation of a respiratory team, frequent round of quarantine wards, employing qualified physicians and nurses, designing common protocols, limiting the hospital stay of patients.

The results of this study showed that the high mortality rate, concern of the nurses’ families, feeling of powerlessness in front of the treatment of the patients is the psychological consequences of covid-19 on the nurses.

Nurses felt overexposed to the virus and were vulnerable to death anxiety, which they experienced through the high COVID-19 mortality rate or their inability to help patients as in previous researches [28]. Study nurses experienced feelings of guilt and inadequacy from not being able to maintain quality of care for their patients. Study nurses perceived their efforts as futile due to the unprecedented number of patient deaths or when they felt patients would no longer benefit from aggressive care [29]. Indeed, nurses were shocked by some of COVID-19 patients’ deaths as they considered they would have been avoidable if there had been no patient prioritization [30].

Findings reveal that most FLWs did not or could not receive any formal training on COVID-19 regarding its prevention and treatment, as well as on the use of except for a few physicians and nurses.

The results of this study showed that nurses did not have scientific information about the quality of PPE, so they used several additional layers of masks that were not approved by the World Health Organization. On the other hand, PPE caused eczema on nurses’ skin and hindered communication with patients, especially elderly patients who had hearing loss.

The FLWs at all levels went through several personal and professional challenges such as shortage of the appropriate and the adequate number of PPE, masks and disinfectants. A study conducted in a tertiary-level hospital in Bangladesh also found that more than 40% of hospital staff had to reuse the PPEs, and only 10% of them had training on PPE [31].

## Conclusion

The results of this study indicated that nurses need more physical, mental, psychological and financial support from their managers. The results of this study can be a basis for the action of hospital managers and nursing managers in order to develop management policies in controlling crises caused by pandemics. As nurses are the largest number of health care workers so, their performance will influence the management of a pandemic like covid-19. Prudent management of critical situations can reduce the rate of physical and mental burnout of nurses. Therefore, this will make nurses more ready and enthusiastic to provide care in future pandemics. As a result of providing high-quality cares by nurses, patient satisfaction will also increase.

### Limitations

Since this study was conducted during the Covid-19 pandemic, accessibility of the nurses for interview was difficult due to the large number of their shifts and fatigue, and to conduct a face-to-face interview. In some cases, the interviews were not completed in one session and were conducted in two sessions. We tried to overcome these limitations by considering the free time of nurses for a long time and increasing the number of sessions in some cases.

### Suggestions

It is suggested that this study be carried out in other countries so that it is possible to extract the best management strategies for patients with covid-19.

## Data Availability

Due to the privacy of the research participants, the data generated during the current study are not publicly available, but are available from the corresponding author upon reasonable request.

## Footnotes

1 . Middle East Respiratory Syndrome Coronavirus.

2 . HCWs.

3 . PPE: personal protective equipment
